# A revised annotated checklist of louse flies (Diptera, Hippoboscidae) from Slovakia

**DOI:** 10.3897/zookeys.862.25992

**Published:** 2019-07-09

**Authors:** Jozef Oboňa, Oldřich Sychra, Stanislav Greš, Petr Heřman, Peter Manko, Jindřich Roháček, Anna Šestáková, Jan Šlapák, Martin Hromada

**Affiliations:** 1 Laboratory and Museum of Evolutionary Ecology, Department of Ecology, Faculty of Humanities and Natural Sciences, University of Presov, 17. novembra 1, SK – 081 16 Prešov, Slovakia University of Presov Prešov Slovakia; 2 Department of Biology and Wildlife Diseases, Faculty of Veterinary Hygiene and Ecology, University of Veterinary and Pharmaceutical Sciences Brno, Palackého tř. 1946/1, CZ – 612 42 Brno, Czech Republic University of Veterinary and Pharmaceutical Sciences Brno Brno Czech Republic; 3 17. novembra 24, SK – 083 01 Sabinov, Slovakia Unaffiliated Sabinov Slovakia; 4 Křivoklát 190, CZ – 270 23, Czech Republic Unaffiliated Křivoklát Czech Republic; 5 Department of Entomology, Silesian Museum, Tyršova 1, CZ-746 01 Opava, Czech Republic Department of Entomology, Silesian Museum Opava Czech Republic; 6 The Western Slovakia Museum, Múzejné námestie 3, SK – 918 09 Trnava, Slovakia he Western Slovakia Museum Trnava Slovakia; 7 Vojtaššákova 592, SK – 027 44 Tvrdošín, Slovakia Unaffiliated Tvrdošín Slovakia; 8 Faculty of Biological Sciences, University of Zielona Gora, Prof. Z. Szafrana 1, 65–516 Zielona Gora, Poland University of Zielona Gora Zielona Gora Poland

**Keywords:** Faunistics, literature review, louse flies, parasite-host associations

## Abstract

The list of all known locality and host records from the literature on louse flies from Slovakia are summarized, with the addition of new collection data. New locality data are provided for five species. Three species are added to the Slovakian list: *Icostaminor* (Bigot in Thomson, 1858), which was erroneously cited for Moravia instead of Slovakia in the previous checklist, and *Ornithophilametallica* (Schiner, 1864) and *Ornithomyachloropus* (Bergroth, 1901), which were overlooked from the last checklist. As a result, the louse fly fauna of Slovakia increases to 19 species: 12 autochtonous species and seven rare, non-native species only occasionally imported to Slovakia or migrating to the country with their hosts. This is by far the largest regional fauna of Hippoboscidae in Central Europe, and matches the richest southern European faunas. In total, 78 host-parasite associations concerning 46 bird-host species from eight orders and nine species of mammals, including humans, have been found from a literature review in Slovakia. Two host-parasite associations are reported from Slovakia for the first time: *Ornithomyaavicularia* (Linnaeus, 1758) on *Prunellamodularis* (Linnaeus, 1758) (Aves: Prunellidae) and *Lipoptenafortisetosa* Maa, 1965 on *Homosapiens* Linnaeus, 1758 (Mammalia: Hominidae).

## Introduction

Flies in the family Hippoboscidae, known as ‘louse flies’ or ‘keds’, belong among the Diptera and are a group of obligate parasites of mammals and birds (Rahola et al. 2011). All species are macrolarviparous, with females retaining the larva in the uterus until the end of the third instar; the three larval instars feed on secretions from the maternal accessory glands. The stage at which larviposition occurs represents a prepupal larva (e.g., Mehlhorn 2016). The larva (or pupa) is deposited in birds’ nests or on the hair of a mammalian host, but sometimes it is deposited on the ground by the female (e.g., Halos et al. 2004). Adults of both sexes are blood sucking and are known to act as vectors of many infectious agents, such as protozoa, bacteria, helminths, and possibly also viruses (e.g., Baker 1967, Kečera 1983, Halos et al. 2004, Liu et al. 2016, Skvarla and Machtinger 2019). Some species are host-specific, whereas others feed on a wide array of hosts (e.g., Ibáñez-Bernal et al. 2015, Mehlhorn 2016, Veiga et al. 2018).

Worldwide, more than 213 louse fly species are known (e.g., Maa 1963, Dick 2006, Rahola et al. 2011): 57 species from the Afrotropical region (e.g., Hutson and Oldroyd 1980, Oboňa et al. 2016), 26 from East Palaearctic Asia and Japan (e.g., Maa 1967, Mogi and Sawada 2002, Matyukhin et al. 2017), 9 from China, 8 from the eastern part of Russia (e.g., Soós and Hůrka 1986, Sun 1999), 6 from the Oriental and Australasian regions (e.g., Paramonov 1954, Amiot and Ji 2015, Farrow 2016), and 31 species have been reported from the Nearctic and Neotropic regions (e.g., Bequaert 1942, 1954, 1965, Savage et al. 2019).

From Europe, 30 species of Hippoboscidae are known (Petersen 2004, Pape et al. 2015). The species composition of the hippoboscid fauna in Slovakia is relatively well known; however, the investigation of Hippoboscidae in Slovakia is still far from complete. Scattered older published data on louse flies by Thalhammer (1899) and Brancsik (1910) are mentioned by Povolný and Rosický (1955). Subsequently, Povolný and Balát (1956), Dyk and Schanzel (1964), Čepelák (1974, 1982, 1985, 1986, 1987, 1988, 1992, 1993, 1994a, 1994b), Čepelák and Čepelák (1991), Chalupský and Macháček (1977), Chalupský (1980, 1986), Krištofík and Štefan (1980), Chalupský and Povolný (1983), Hubálek et al. (1988), Krištofík (1998), Kočišová et al. (2007), Roháček (1995, 2009), Straka (1981, 2001, 2005a, 2005b, 2010, 2011, 2016), Straka and Majzlán (2008, 2010, 2014, 2016), and Kočišová (2015) published additional information on the hippoboscid fauna of Slovakia. The most recent checklist of the family Hippoboscidae from Slovakia (Sychra 2009) comprised 16 species.

## Materials and methods

Samples of Hippoboscini, Lipoptenini, and Ornithomyini came from unidentified material in the collections of the Laboratory and Museum of Evolutionary Ecology, Department of Ecology, University of Presov (LMEE PO); of the Department of Entomology, Silesian Museum, Opava (SMOC); and of the Department of Biology and Wildlife Diseases, Faculty of Veterinary Hygiene and Ecology, University of Veterinary and Pharmaceutical Sciences Brno (VFU).

The material was identified using Povolný and Rosický (1955), Chalupský and Povolný (1983), Hutson (1984), Ducháč and Bádr (1998). The systematics and nomenclature follow Hutson and Oldroyd (1980), Kutty et al. (2010), Petersen et al. (2007), and Evenhuis et al. (2016).

## Results

### Tribe Hippoboscini

***Hippoboscaequina* Linnaeus, 1758**

**Published records**: Ladomirov (Ladomér), Rožňava (Rozsnyó), Slovenské Nové Mesto (S.A. Ujhely), Stropkov (Sztropkó – Olyka) (Thalhammer 1899); Trenčín (Trencsén) (Brancsik 1910, Chalupský 1986); Kečovo, Remetské Hámre, Rožňava (Povolný and Rosický 1955, Chalupský 1986); Bratislava, Kurinec, Staré Hory, Šaštín – Stráže, Veľké Leváre (Krištofík and Štefan 1980, Chalupský 1986); Kráľovský Chlmec, Plešivecká planina (plain), Silica, Zádiel (Chalupský and Povolný 1983); Chľaba (Čepelák 1986); Dolné Štitáre, Svorad, Žibrica (Čepelák and Čepelák 1991); Veľký Lysec (Čepelák 1992); Hunták (Čepelák 1993); Krivín (Čepelák 1994b); Bratislava-Lamač, Devínska Nová Ves, Horné Orešany, Jakubov, ostrov Kopač (island), Malacky, Štúrovo (Krištofík 1998); “Veľká Fatra” (Straka 2005b); Bábsky les (forest) (Straka and Majzlán 2010); Obručná, Radzovce (Straka 2016); Burdov, Leliansky les (forest) (Straka and Majzlán 2016).

**Published host records**: main host: *Equuscaballus* Linnaeus, 1758 (see Povolný and Rosický 1955, Krištofík and Štefan 1980, Chalupský and Povolný 1983, Krištofík 1998); occasional hosts: *Accipitergentilis* (Linnaeus, 1758) (see Krištofík and Štefan 1980), *Bostaurus* Linnaeus, 1758 (see Povolný and Rosický 1955, Chalupský and Povolný 1983), *Canisfamiliaris* Linnaeus, 1758 (see Povolný and Rosický 1955), *Capreoluscapreolus* (Linnaeus, 1758) (see Povolný and Rosický 1955, Chalupský and Povolný 1983), *Homosapiens* Linnaeus, 1758 (see Povolný and Rosický 1955, Krištofík and Štefan 1980, Krištofík 1998).

**Figure 1.**
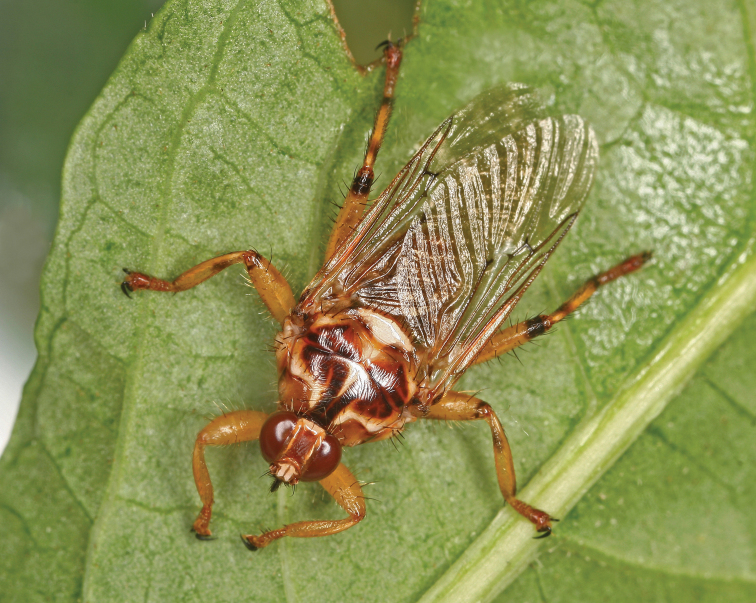
Female of *Hippoboscaequina* Linnaeus, 1758 from Muránska planina NP (J Roháček).

**Material examined**: Báb, 48°18'21.6"N, 17°53'16.5"E, 150 m a.s.l., 11.6.2007, 1 female, malaise trap, O. Majzlán leg. (LMEE PO); Drienovec, 48°37'04.4"N, 20°55'29.9"E, 200 m a.s.l., 1.10.2015, 1 female, from human, S. Greš leg. (LMEE PO); Kamenica nad Hronom nr. Štúrovo, 47°49'30"N, 18°43'03"E, 105 m a.s.l., 17.5.1984, 1 female, J. Roháček leg. (SMOC); Muránska planina NP, Poludnica res.-Suchý dol, 48°45'26"N, 20°02'32"E, 480 m a.s.l., 6.9.2011, 1 female (Fig. 1), sweeping over pasture meadow, J. Roháček leg. (SMOC); Muránska planina NP, Muráň castle env., 48°45'03"N, 20°02'54"E, 625 m a.s.l.; 4.5.2015, 1 male, sweeping undergrowth of steppe forest, J. Roháček leg. (SMOC); Cerová vrchovina PLA, Gemerský Jablonec – Vodokáš 1 km N, 48°13'00"N, 19°59'42"E, 280 m a.s.l., 6.9.2017, 1 male, sweeping over steppe meadow, J. Roháček leg. (SMOC); Cerová vrchovina PLA, Tachty 2.2 km SW, Gortva valley, 48°08'41"N, 19°54'51"E, 320 m a.s.l., 13.9.2018, 1 female, netted from forest margin vegetation, J. Roháček leg. (SMOC).

**Comments**: *H.equina* is a Palaearctic and West Oriental species. It is relatively large, once common, presently disappearing from Central Europe. An ectoparasite of livestock (preferably horses and donkeys) and dogs, but also attacks humans (Krištofík 1998). Previously published Slovak records are from the years 1953–1977, with a single record from 2007 (Straka and Majzlán 2010). Its recent occurrence is strongly affected by a decline of traditional horse and sheep farming in the monitored area (e.g., Bezák and Petrovič 2006). Classified as endangered (EN) in Slovakia (Jedlička and Stloukalová 2001).

***Hippoboscalongipennis* Fabricius, 1805**

**Published records**: Kečovo (Chalupský 1980, Chalupský and Povolný 1983, Chalupský 1986); Bábsky les (Straka and Majzlán 2010).

**Published host records**: *Canisfamiliaris* (see Chalupský 1980, Chalupský and Povolný 1983).

**Comments**: A rare and non-native species in Central Europe, distributed in the Mediterranean and Afrotropical regions. Up until now, only two individuals have been recorded in Slovakia, in 1953 and 2007 (Chalupský 1980, Straka and Majzlán 2010). It is an ectoparasite of dogs; occasionally it can occur also on other predatory mammals or ungulates (Chalupský 1980). Occassionally, it can be imported via human activities (e.g., through breeding of particular species of mammal).

***Hippoboscavariegata* Megerle, 1803**

**Published records**: Gabčíkovo (Povolný and Rosický 1955, as *H.maculata*, Chalupský 1980, Chalupský 1986).

**Published host records**: *Canisfamiliaris* (see Povolný and Rosický 1955, Chalupský 1980).

**Comments**: A rare and non-native species in Central Europe, distributed in the Afrotropical region. The only record from Slovakia is from 1951. It is an ectoparasite of cattle and domestic equines (Povolný and Rosický 1955). It can be occasionally imported through human activities (e.g., through breeding of particular species of mammals).

### Tribe Lipoptenini

***Lipoptenacervi* (Linnaeus, 1758)**

**Published records**: Mníchova Lehota (Barátszabadi), Omšenie (Nagysziklás) (Brancsik 1910); Dlhá Ves, Timoradz (Povolný and Rosický 1955); Blatnická dolina (valley in Veľká Fatra) (Dyk and Schanzel 1964); Chľaba, Hlboké, Jovsa, Kamienka, Kúty, Lozorno (Krištofík and Štefan 1980, Chalupský 1986); Gabčíkovo, Modrá, Nitra, Plášťovce, Plešivec, Podunajské Biskupice, Silická planina (plain), Šaštín – Stráže, Timoradza (Chalupský and Povolný 1983, Chalupský 1986); Burdov (Čepelák 1986); Topoľčany, (Chalupský 1986); Hrášková Lúka (Čepelák 1987); Hrdovická (Čepelák 1988); Silická planina (plain) (Hubálek et al. 1988); Bartošov prameň (well), Dolné Štitáre, Gáborka, Haranč, Hrnčiarovce, Hunták (Čepelák and Čepelák 1991); Veľký Lysec (Čepelák 1992); Nebrová (Čepelák 1994a); Nové Mesto nad Váhom, Podunajské Biskupice, Stará Lesná, Šaštín – Stráže, Veľká Fatra – Gaderská dolina (valley) (Krištofík 1998); Klín, Rozsutec (Straka 2001); Hrochoť – Beňova dolina (valley) (Roháček 2009); Žalostiná (Straka 2010); Vršatské bradlá (cliff), Záhorská Bystrica (Straka 2011); “Nitrické vrchy” (Straka and Majzlán 2014).

**Published host records**: main hosts: *Capreoluscapreolus* (see Dyk and Schanzel 1964, Krištofík and Štefan 1980, Chalupský and Povolný 1983, Krištofík 1998); *Cervuselaphus* Linnaeus, 1758 (see Dyk and Schanzel 1964, Krištofík and Štefan 1980, Chalupský and Povolný 1983, Hubálek et al. 1988); occasional hosts: *Caprahircus* Linnaeus, 1758 (see Povolný and Rosický 1955); *Homosapiens* (see Krištofík and Štefan 1980, Krištofík 1998); *Rupicaprarupicapra* (Linnaeus, 1758) (see Krištofík 1998); *Turdusphilomelos* C. L. Brehm, 1831 (see Chalupský and Povolný 1983).

**Figure 2.**
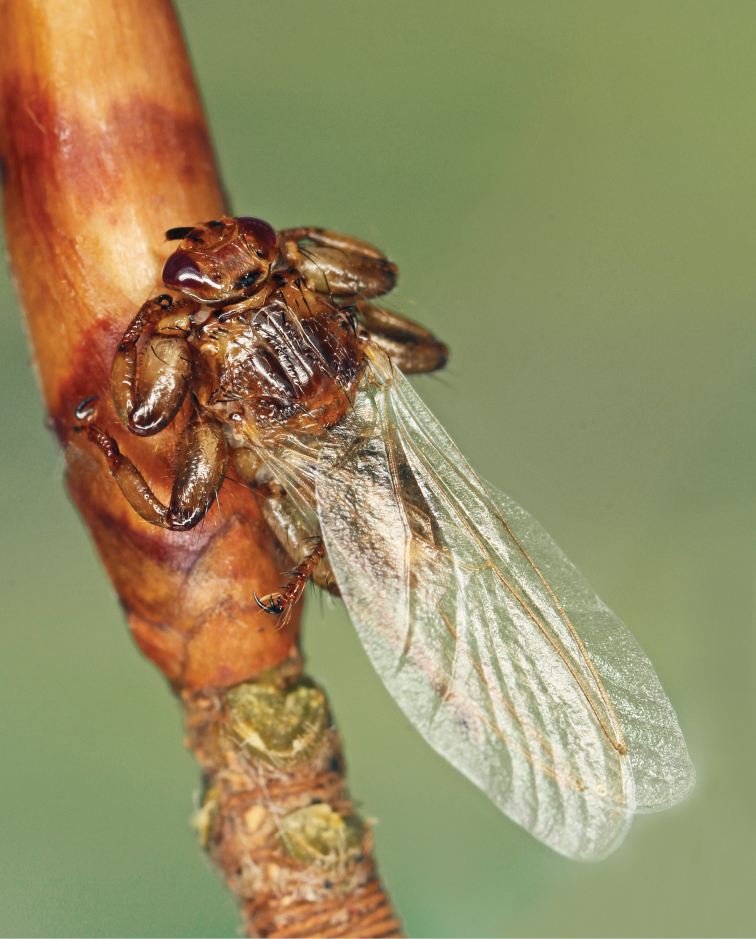
Female of *Lipoptenacervi* (Linnaeus, 1758) from Cerová vrchovina PLA (J Roháček).

**Material examined**: Diviacka Nová Ves, 48°44'58.9"N, 18°29'29.5"E, 280 m a.s.l., 4.9.2012, 1 male, from a human, J. Oboňa leg. (LMEE PO); Drienovec, 48°37'04.4"N, 20°55'29.9"E, 200 m a.s.l., 1.10.2015, 2 males, 1 female, from human, S. Greš leg. (LMEE PO); Stará Lesná, 49°08'11.3"N, 20°17'47.5"E, 750 m a.s.l., 8.9.2017, 1 male, from human, P. Manko leg. (LMEE PO); Tvrdošín (Skorušické vrchy), 49°22'19.5"N, 19°31'57.4"E, 750 m a.s.l., 23.9.2017, 1 female, from human, J. Šlapák leg. (LMEE PO); Východná, 49°04'04.2"N, 19°53'57.0"E, 780 m a.s.l., 15.9.2017, 1 male, from car, A. Šestáková leg. (LMEE PO); Nová Sedlica env., 49°03'22.1"N, 22°31'03.1"E, 505 m a.s.l., 1.10.1997, 4 females, sweeping undergrowth of deciduous forest, J. Roháček leg. (SMOC); Muránska planina NP, Šiance res., top plateau, 48°46'11"N, 20°04'14"E, 1000 m a.s.l., 7.9.2011, 1 male, the same, 4.9.2012, 1 male, J. Roháček leg. (SMOC); Muránska planina NP, Pohronská Polhora 5.9 km E, Kučalach Mt., 48°44'51"N, 19°52'27"E, 1060 m a.s.l., 10.10.2014, 2 females, sweeping undergrowth of beech-fir forest, J. Roháček leg. (SMOC); Muránska planina NP, Šarkanica res., 48°42'45"N, 19°59'19"E, 580 m a.s.l., 29.9.2017, 1 female, sweeping undergrowth of deciduous forest in ravine, J. Roháček leg. (SMOC); Cerová vrchovina PLA, Gemerský Jablonec – Vodokáš 1 km N, 48°13'00"N, 19°59'42"E, 280 m a.s.l., 27.9.2017, 1 male, the same, 1.11.2017, 3 females, sweeping undergrowth of oak-beach forest (Fig. 2), J. Roháček leg. (SMOC).

**Comments**: A relatively frequent species in Central Europe, widespread in the Palaearctic region and introduced to the Nearctic region. It is an ectoparasite of Cervidae, and also attacks human beings (Krištofík 1998).

***Lipoptenafortisetosa* Maa, 1965**

**Published records**: Rozhanovce (Kočišová et al. 2007).

**Published host records**: *Capreoluscapreolus* (Kočišová et al. 2007).

**Material examined**: Bogliarka, 49°16'37.1"N, 21°08'52.3"E, 450 m a.s.l., 15.7.2017, 1 female, from human, P. Manko leg. (LMEE PO); Lažany, 49°02'20.2"N, 21°05'40.8"E, 380 m a.s.l., 7.2017, 17 males, 13 females, 21.6.2017, 1 female, 5.9.2017, 4 males, 2 females, all from human, P. Manko leg. (LMEE PO); Levočská (dolina) valley, 49°04'08.3"N, 20°36'17.5"E, 780 m a.s.l., 12.8.2017, 1 female, from a human, A. Šestáková leg. (LMEE PO); Magurka – Oravská Magura (Oravská priehrada), 49°23'19.6"N, 19°31'34.3"E, 850 m a.s.l., 29.7.2017, 1 male, 1 female, 650 m a.s.l., 17.7.2017, 1 male, all from human, J. Šlapák leg. (LMEE PO); Prešov env. (near “pri Kríži”), 48°59'57.0"N, 21°13'03.7"E, 300 m a.s.l., 9.9.2017, 1 male, from a human, J. Oboňa leg. (LMEE PO); Stráne pod Tatrami, 49°09'26.7"N, 20°21'59.9"E, 750 m a.s.l., 8.9.2017, 1 male, from a human, J. Oboňa leg. (LMEE PO); Tvrdošín (Skorušické vrchy), 49°22'19.5"N, 19°31'57.4"E, 700 – 750 m a.s.l., 7.2016, 1 female, 10.7.2017, 1 male, 22.7.2017, 1 male, 3 females, 26.7.2017, 1 male, 31.8.2017, 1 female, all from human, J. Šlapák leg. (LMEE PO); Cerová vrchovina PLA, Tachty 2.2 km SW, Gortva valley, 48°08'41"N, 19°54'51"E, 320 m a.s.l., 13.9.2018, 2 females, netted from forest margin vegetation, J. Roháček leg. (SMOC).

**Comments**: A relatively frequent species in Slovakia, distributed in the eastern Palaearctic region. Previously often confused with *Lipoptenacervi*. *L.fortisetosa* has a western boundary of distribution in Central Europe. It is an ectoparasite of Cervidae, and also attacks human beings (Ducháč and Bádr 1998). *Homosapiens* is here newly recorded as a (common) host of *L.fortisetosa* in Slovakia.

***Melophagusovinus* (Linnaeus, 1758)**

**Published records**: Štúrovo (Povolný and Rosický 1955); Silica (Povolný and Rosický 1955, Hubálek et al. 1988, Chalupský 1986); Hažín, Kôprová dolina (valley), Šahy (Chalupský and Povolný 1983, Chalupský 1986); Poprad (Kočišová 2015).

**Published host records**: main host: *Ovisaries* Linnaeus, 1758 (see Povolný and Rosický 1955, Chalupský and Povolný 1983, Hubálek et al. 1988); occasional hosts: *Canisfamiliaris* and *Homosapiens* (see Povolný and Rosický 1955), and *Equuscaballus* (Kočišová 2015).

**Comments**: It is an ectoparasite of Bovidae, especially sheep (including lambs) (Chalupský and Povolný 1983). *Melophagusovinus* is strongly affected by a decline of traditional horse and sheep farming in the monitored area (e.g., Bezák and Petrovič 2006).

***Melophagusrupicaprinus* Rondani, 1879**

**Published records**: Námestovo (Chalupský 1980, Chalupský 1986); Tatranská Kotlina (Chalupský and Povolný 1983, Chalupský 1986).

**Published host records**: *Ovisaries* (see Chalupský 1980), *Rupicaprarupicapra* (see Chalupský and Povolný 1983).

**Comments**: A relatively rare species in Central Europe. In Slovakia it is listed as endangered (EN) (Jedlička and Stloukalová 2001). Until now, only two records from 1951 and 1980 have been published from Slovakia (Chalupský 1980, Chalupský and Povolný 1983). It is an ectoparasite of mammals, collected mainly from *R.rupicapra* and, secondarily, sheep (Chalupský and Povolný 1983). *Rupicaprarupicapra* has an isolated population (R.rupicaprassp.tatrica) in the Tatra Mountains in the north of the country, where *M.rupicaprinus* are found. Because it is isolated at the edge of its distribution range, this population of *M.rupicaprinus* is very vulnerable and like many other marginal populations, it could disappear rapidly and suddenly.

### Tribe Olfersiini

***Crataerinapallida* (Olivier in Latreille, 1811)**

**Published records**: no localities (Povolný and Rosický 1955); Banská Bystrica (Krištofík and Štefan 1980, Chalupský 1986); Vrútky (Straka 1981); Suchý (Čepelák 1985, Chalupský 1986); Bratislava (Krištofík 1998).

**Published host records**: *Apusapus* (Linnaeus, 1758) (see Povolný and Rosický 1955, Krištofík and Štefan 1980, Straka 1981, Krištofík 1998).

**Comments**: A frequent louse fly species in Central Europe, widespread in the Palaearctic region. A common ectoparasite of the bird species *Apusapus*, *Delichonurbicum* (Linnaeus, 1758) and (infrequently) of species from other birds, most frequently on young individuals (Krištofík 1998).

Even if the hosts are still widespread, their population density has declined over the past decades, and therefore the parasites will also suffer (BirdLife International 2018).

***Icostaardeae* (Macquart, 1835)**

**Published records**: Boheľov (Krištofík and Štefan 1980, Chalupský 1986).

**Published hosts**: *Ardeapurpurea* Linnaeus, 1766 (Krištofík and Štefan 1980), *Ixobrychusminutus* (Linnaeus, 1766) (Krištofík and Štefan 1980).

**Comments**: A relatively rare species in Central Europe, widespread in the tropics and subtropics of the Old World. In Slovakia it is listed as vulnerable (VU) (Jedlička and Stloukalová 2001). Until now, only two records from 1977 have been published from Slovakia. *Icostaaredeae* is an ectoparasite of birds belonging to several different families; it is common on Ardeidae, and rarely found on species from other families (Krištofík and Štefan 1980). The host *Ardeapurpurea* is mainly found in the SW part of the country, and although *Ixobrychusminutus* has a wider distribution, it is also more common in SW Slovakia. The rarity of this parasite in Slovakia is mainly due to its occurrence on the edge of its range (and hosts’ ranges) in the country.

***Icostaminor* (Bigot in Thomson, 1858)**

**Published records**: Patince (Chalupský and Macháček 1977, Chalupský 1986).

**Published host records**: *Passermontanus* (Linnaeus, 1758) (see Chalupský and Macháček 1977).

**Comments**: A relatively small, rare and non-native species in Central Europe, distributed in the Afrotropical region and the Mediterranean Basin. In Slovakia it was erroneously listed as vulnerable (VU) (Jedlička and Stloukalová 2001), even though it is not a native species in the country. An ectoparasite on various species of Passeriformes, less frequently found on species from other bird orders (Chalupský and Macháček 1977). The only known record from Slovakia is from 1974 (Chalupský and Macháček 1977, Chalupský 1980). It was mistakenly cited as occurring in the Czech Republic by Chalupský and Povolný (1987, 1997) and Sychra (2006, 2009).

***Olfersiafumipennis* (Sahlberg, 1886)**

**Published records**: “Slovakia” (Povolný and Balát 1956, as *Lynchiapalustris*, Chalupský 1986).

**Published host records**: “eagle” (Povolný and Balát 1956).

**Comments**: A rare and non-native species in Central Europe, distributed mainly in the Nearctic and Neotropical regions. It is an ectoparasite of birds, mainly birds of prey (Chalupský 1980). The only known record from Slovakia is from 1904. That individual was originally misidentified as *Ornithophilametallica* by A. Wimmer (see Povolný and Balát 1956). Also, Povolný and Balát (1956) mentioned this specimen mistakenly under the name *Lynchiapalustris*, which is in fact a synonym of *Icostaalbipennis* from America (Chalupský 1980).

***Ornithoicaturdi* (Olivier in Latreille, 1811)**

**Published records**: Košice, Podunajské Biskupice (Povolný and Rosický 1955, Chalupský 1986); Podunajské Biskupice (Chalupský 1980, Chalupský and Povolný 1983).

**Published host records**: *Emberizacitrinella* Linnaeus, 1758 (see Povolný and Rosický 1955); *Fringillacoelebs* Linnaeus, 1758 and *Sittaeuropaea* Linnaeus, 1758 (see Povolný and Rosický 1955, Chalupský and Povolný 1983).

**Comments**: A relatively small species distributed in the Afrotropical region and southern Palaearctic, with a recent increase in records from Central Europe (Droz and Haenni 2011). In Slovakia, where it reaches the northernmost limit of its known distribution, it has been recorded only from a few individuals collected in 1953 (Povolný and Rosický 1955, Chalupský and Povolný 1983), and it was listed as vulnerable (VU) by Jedlička and Stloukalová (2001). It is an ectoparasite of birds, mainly small Passeriformes. It is less frequently found on species from other bird orders (Krištofík 1998).

***Ornithophilametallica* (Schiner, 1864)**

**Published records**: Jakubov (Krištofík 1998).

**Published host records**: *Saxicolarubetra* (Linnaeus, 1758) (see Krištofík 1998).

**Comments**: A rare and non-native species in Central Europe, distributed in southern parts of the Palaearctic, Afrotropical, Oriental and Australasian regions. The only known record from Slovakia is from 1993 (Krištofík 1998). It was, however, overlooked and not listed in the most recent checklist (Sychra 2009). It is an ectoparasite of birds, mainly small Passeriformes but also species from other bird orders (Krištofík 1998).

***Pseudolynchiacanariensis* (Macquart in Webb & Berthelot, 1839)**

**Published records**: Devín (Povolný and Balát 1956, Chalupský 1986)

**Published host records**: *Pandionhaliaetus* (Linnaeus, 1758) (Povolný and Balát 1956, Chalupský 1980).

**Comments**: A relatively rare and non-native species in Central Europe, widespread (subcosmopolitan) in the tropical and subtropical belts. In Slovakia it has been listed as vulnerable (VU) (Jedlička and Stloukalová 2001), despite not being a native species. An ectoparasite on species of many bird families but preferentially associated with Columbidae, including the domestic pigeon (Chalupský 1980). The only known record from Slovakia is from 1949 (Povolný and Balát 1956).

***Stenepteryxhirundinis* (Linnaeus, 1758)**

**Published records**: Vyhne (Vihnye) (Thalhammer 1899); Trenčín (Trencsén) (Brancsik 1910, Chalupský 1986); Devínska Nová Ves (Povolný and Rosický 1955, Chalupský 1986); Bratislava (Krištofík 1998).

**Published host records**: *Delichonurbicum* (see Povolný and Rosický 1955; Krištofík 1998); *Hirundorustica* Linnaeus, 1758 (see Thalhammer 1899).

**Comments**: A frequent Central European species, widespread in the Palaearctic region. A common ectoparasite of the bird species *Delichonurbicum*, *Hirundorustica*, *Ptyonoprognerupestris* (Scopoli, 1769), *Ripariariparia* (Linnaeus, 1758), and (more rarely) of species from other bird species, most frequently found in nests (Krištofík 1998). *Stenepteryxhirundinis* might suffer from the decline of its hosts (BirdLife International 2018).

### Tribe Ornithomyini

***Ornithomyaavicularia* (Linnaeus, 1758)**

Fig. 3

**Published records**: Snina (Szinna) (Thalhammer 1899); Súľov (Čepelák 1974), Bratislava, Čalovec, Číčov, Jarok, Lozorno, Plešivec, Sása (Krištofík and Štefan 1980, Chalupský 1986); Kečovo, Šurany, Vtáčnik (Povolný and Rosický 1955, Chalupský and Povolný 1983, Chalupský 1986); Dražovce, Nitra (Čepelák and Čepelák 1991); Rača (Čepelák 1982, Chalupský 1986); Ivánka pri Dunaji, Nitra, Rača, Sládkovičovo (Chalupský 1986); Uličské Krivé (Roháček 1995); Bratislava, Brzotín, Gbelce, Kiarov, Kňažia, Kostolište, Košice – Šaca, Košická Nová Ves, Limbach, Mojš, Oravský Podzámok, Pavlovce nad Váhom, Pezinok, Plavecký Mikuláš, Podunajské Biskupice, Svätý Jur, Šiatorská Bukovinka, Závod (Krištofík 1998); Bábsky les (Straka and Majzlán 2010).

**Figure 3.**
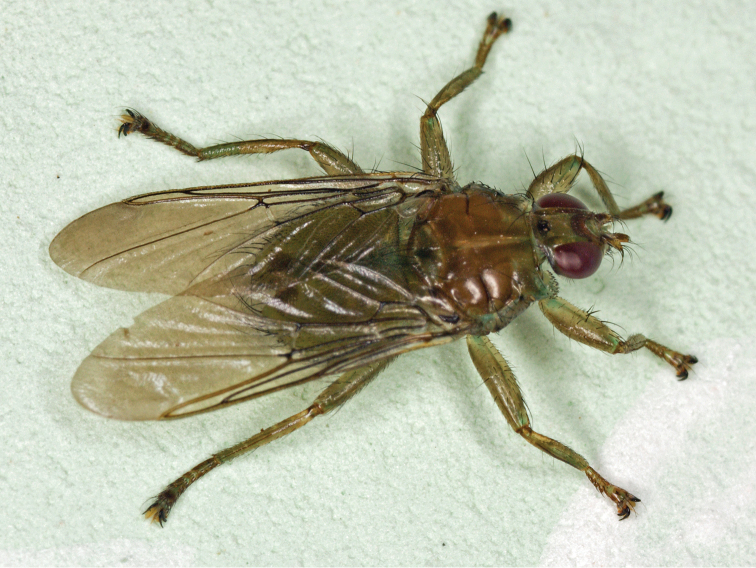
Female of *Ornithomyaavicularia* (Linnaeus, 1758) (M Deml).

**Published host records**: *Accipitergentilis* (Linnaeus, 1758), *Acrocephalusarundinaceus* (Linnaeus, 1758), *A.melanopogon* (Temminck, 1823), *A.scirpaceus* (Hermann, 1804), *Anseranser* (Linnaeus, 1758) (see Krištofík 1998); *Anthustrivialis* (Linnaeus, 1758) (see Krištofík and Štefan 1980; Krištofík 1998); *Aquilapomarina* C. L. Brehm, 1831 (see Krištofík 1998); *Carduelischloris* (Linnaeus, 1758) (see Chalupský and Povolný 1983); *Circuspygargus* (Linnaeus, 1758), *Coccothraustescoccothraustes* (Linnaeus, 1758), *Emberizacia* Linnaeus, 1766, *Erithacusrubecula* (Linnaeus, 1758) (see Krištofík 1998); *Falcotinnunculus* Linnaeus, 1758 (Krištofík and Štefan 1980); *Ficedulaalbicollis* (Temminck, 1815), *Fringillacoelebs* Linnaeus, 1758 (see Krištofík 1998); *Homosapiens* (Krištofík and Štefan 1980); *Laniuscollurio* Linnaeus, 1758, *L.excubitor* Linnaeus, 1758, *Locustellaluscinioides* (Savi, 1824), *Monticolasaxatilis* (Linnaeus, 1766), *Oriolusoriolus* (Linnaeus, 1758), *Panurusbiarmicus* (Linnaeus, 1758) (see Krištofík 1998); *Passerdomesticus* (Linnaeus, 1758) (see Krištofík and Štefan 1980); *Passermontanus* (Linnaeus, 1758) (see Krištofík 1998); *Pernisapivorus* (Linnaeus, 1758) (see Thalhammer 1899); *Phoenicurusochruros* (S. G. Gmelin, 1774), *Picapica* (Linnaeus, 1758) (see Krištofík 1998); *Picuscanus* J. F. Gmelin, 1788 (see Chalupský and Povolný 1983); *Saxicolarubetra* (Linnaeus, 1758) (see Krištofík 1998); *Strixaluco* Linnaeus, 1758 (see Chalupský and Povolný 1983); *Turdusmerula* Linnaeus, 1758 (see Čepelák and Čepelák 1991; Krištofík 1998); *Turduspilaris* Linnaeus, 1758 (see Krištofík 1998).

**Material examined**: Diviacka Nová Ves, 48°44'58.9"N, 18°29'29.5"E, 280 m a.s.l., 15.8.2012, 1 female, on a family house, J. Oboňa leg. (LMEE PO); Drienovec, 48°37'04.4"N, 20°55'29.9"E, 200 m a.s.l., 15.9.2015; 1 female, from *Prunellamodularis* (Linnaeus, 1758), S. Greš leg. (LMEE PO).

**Comments**: A frequent louse fly species in Central Europe, widespread in the Palaearctic region. A common ectoparasite of birds from the order Passeriformes and other orders, most frequently find in more individuals per host (Krištofík 1998). *Prunellamodularis* is here recorded as a new host of *O.avicularia* in Slovakia.

***Ornithomyabiloba* Dufour, 1827**

**Published records**: Omšenie (Nagysziklás) (Brancsik 1910, as *Ornithomyiatenella*); Čaradská pustatina (Krištofík and Štefan 1980, Chalupský 1986); Humenné (Chalupský and Povolný 1983, Chalupský 1986); Bašovce, Bernolákovo, Bodíky, Kľúčovec, Podunajské Biskupice (Krištofík 1998).

**Published host records**: *Hirundorustica* (see Krištofík 1998, Krištofík and Štefan 1980); *Ripariariparia* (see Chalupský and Povolný 1983).

**Material examined**: Gbelce, 47°51'29.4"N, 18°30'17.9"E, 120 m a.s.l., 21.4.2009, 1 male, 27.4.2009, 1 male, 28.4.2009, 1 male, 2.5.2009, 2 males, all from *Hirundorustica*, O. Sychra leg. (VFU).

**Comments**: A Palaearctic species, common in Central Europe; its distribution range is incompletely known. It is an ectoparasite mainly of *Delichonurbicum*, *Hirundorustica*, *Ripariariparia* and, less often, of species from other bird orders (Krištofík 1998).

***Ornithomyachloropus* (Bergroth, 1901)**

**Published records**: Kalinovo, Košice (Povolný and Rosický 1955, Chalupský 1986).

**Published host records**: *Regulusregulus* (Linnaeus, 1758) (see Povolný and Rosický 1955); without host record (Chalupský 1986).

**Comments**: A Palaearctic species distributed in the northern and middle belts of the region. It is an ectoparasite mainly of Passeriformes, but also of species of other bird orders (Povolný and Rosický 1955). The only known record from Slovakia is from 1953, and it was “hidden” in the figure legend in Povolný and Rosický (1955). It was incorrectly doubted by Chalupský (1980) and was not listed in the last (Sychra 2009) or all previous checklists (Chalupský and Povolný 1987, 1997, Sychra 2006).

***Ornithomyafringillina* Curtis, 1836**

**Published records**: Boheľov, Kamienka, Pilsko, Rovinka (Krištofík and Štefan 1980, Chalupský 1986); Kalinovo (Chalupský and Povolný 1983, Chalupský 1986); Brodské, Číčov, Gbelce, Jakobov, Oravský Podzámok, Svätý Jur (Krištofík 1998); Krasín (Straka 2005a); Lutovský Drieňovec (Straka and Majzlán 2008); “Nitrické vrchy” (Straka and Majzlán 2014).

**Published host records**: *Acrocephalusarundinaceus*, *A.schoenobaenus* (Linnaeus, 1758) (see Krištofík 1998); *Ardeapurpurea* (see Krištofík and Štefan 1980); *Paruscaeruleus* Linnaeus, 1758 (see Krištofík and Štefan 1980; Krištofík 1998); *Parusmajor* Linnaeus, 1758, *Ripariariparia* (see Krištofík and Štefan 1980); *Sittaeuropaea*, *Sylviaatricapilla* (Linnaeus, 1758) (see Krištofík 1998); *Troglodytestroglodytes* (Linnaeus, 1758) (see Krištofík and Štefan 1980).

**Comments**: A Palaearctic species distributed in the northern and middle belts of the region. It is an ectoparasite mainly of Passeriformes, but also parasitizes species of other bird orders (Krištofík 1998).

## Discussion

We have critically evaluated all available data on the occurrence of the family Hippoboscidae in Slovakia, and published data are completed with new collection data and unpublished localities. We confirmed 19 species as recorded from the country, which has one of the richest hippoboscid faunas in Europe. Out of 19 total species, 12 are native. While seven species (*Crataerinapallida*, *Lipoptenacervi*, *L.fortisetosa*, *Ornithomyaavicularia*, *O.biloba*, *O.fringillina*, and *Stenepteryxhirundinis*) are widespread, three species (*Icostaardeae*, *Melophagusrupicaprinus*, and *Ornithomyachloropus*) are known only from a few records, and the occurrence of the last two species (*Hippoboscaequina* and *Melophagusovinus*) is strongly affected by a decline of traditional horse and sheep farming in the monitored area (e.g., Bezák and Petrovič 2006). Species *S.hirundinis* and *C.pallida* might suffer from the decline of its hosts (BirdLife International 2018). Another seven species (*Hippoboscalongipennis*, *H.variegata*, *Icostaminor*, *Olfersiafumipennis*, *Ornithoicaturdi*, *Ornithophilametallica*, and *Pseudolynchiacanariensis*) have been recorded from Slovakia based on very few records, due to occasional introduction with their hosts. These species can be introduced naturally due to migrating hosts (e.g., *Icostaminor*, *Olfersiafumipennis*, *Ornithophilametallica*, and *Pseudolynchiacanariensis*) or imported together with domestic animals (e.g., *Hippoboscalongipennis*, *H.variegata*).

Previous records of three species were omitted from the most recent checklist (Sychra 2009):

1) a single record of *Icostaminor* from Slovakia (Chalupský 1980; Chalupský and Macháček 1977) had been erroneously cited as being from Moravia (Czech Republic) in previous checklists (Chalupský and Povolný 1987, 1997; Sychra 2006, 2009);

2) *Ornithophilametallica* was reported from Slovakia by Krištofík (1998), and

3) *Ornithomyachloropus* (Bergroth, 1901) was reported from Slovakia by Povolný and Rosický (1955) in a note “hidden” in the illustration legend, but these records were omitted from all versions of the regional checklist (Chalupský and Povolný 1987, 1997; Sychra 2006, 2009), possibly due to Chalupský (1980), who doubted its occurrence in Slovakia.

Altogether, 78 host-parasite associations have so far been recorded for Slovakian Hippoboscidae (Table 2). The hosts of the 19 species of louse flies recorded in Slovakia belong to 46 species of birds from eight orders (Accipitriformes, Anseriformes, Apodiformes, Ciconiiformes, Falconiformes, Passeriformes, Piciformes, Strigiformes) and nine species of mammals, including humans. The host records of *Prunellamodularis* for *O.avicularia* and *Homosapiens* for *L.fortisetosa* are here recorded from Slovakia for the first time.

The species composition of the hippoboscid fauna of Slovakia is relatively well known, and is, in comparison with other European countries, unexpectedly diverse. In Table 1, a list of European hippoboscid faunas is given, based on Petersen (2004) and Pape et al. (2015) and supplemented with data from relatively recent regional checklists (Büttiker 1998, Chandler 1998, Müller 1999, Beuk 2001, Draber-Monko 1991, Pape et al. 1995, Papp 2001, Carles-Tolrá and Báez 2002, Sychra 2009, Pohjoismäki and Kahanpää 2014, present paper).

**Table 1.** Hippoboscid faunas of selected European countries (see Petersen 2004, Pape et al. 2015), supplemented with data from relative recent checklists.^*^

			**Spain inc. islands**	**Italy inc. islands**	**Switzerland**	**Great Britain**	**Czech Republic**	**Slovakia**	**Finland**	**Germany**	**Hungary**	**Poland**	**The Netherlands**
Hippoboscini	* Hippobosca *	* equina *	+	+	+	+	+	+	+	+	+	+	+
		* longipennis *	+	+		+		+			+		
		* variegata *						+					
Lipoptenini	* Lipoptena *	* arianae *											
		* capreoli *											
		* cervi *	+	+	+	+	+	+	+	+	+	+	+
		* couturieri *	+										
		* fortisetosa *			+		+	+		+		+	
	* Melophagus *	* ovinus *	+	+	+	+	+	+	+	+	+	+	+
		* rupicaprinus *		+	+			+		+			
Olfersiini	* Crataerina *	* acutipennis *	+										
		* melbae *	+	+	+								
		* obtusipennis *											
		* pallida *	+	+	+	+	+	+	+	+	+	+	+
	* Icosta *	* ardeae *		+	+	+	+	+			+		+
		* massonati *											
		* minor *	+	+		+		+					
	* Olfersia *	* fumipennis *	+				+	+	+				
		* spinifera *				+							
	* Ornithoica *	* turdi *	+	+			+	+			+		+
	* Ornithophila *	* gestroi *	+	+									
		* metallica *	+	+	+	+	+	+	+	+		+	+
	* Pseudolynchia *	* canariensis *	+	+			+	+					
		* garzettae *		+		+							
	* Stenepteryx *	* hirundinis *	+	+	+	+	+	+	+	+	+	+	+
Ornithomyiini	* Ornithomya *	* avicularia *	+	+	+	+	+	+	+	+	+	+	+
		* biloba *		+	+	+	+	+		+	+	+	+
		* chloropus *	+	+	+	+	+	+	+	+	+	+	+
		* fringillina *	+	+	+	+	+	+	+	+	+	+	+
		* rupes *	+		+								
**number of species**			19	19	15	15	15	19	10	12	12	11	12

^*^ Spain incl. islands (Carles-Tolrá and Báez 2002), Italy incl. islands (Pape et al. 1995), Switzerland (Büttiker 1998), Great Britain (Chandler 1998), Czech Republic and Slovakia (Sychra 2009, present paper), Finland (Pohjoismäki and Kahanpää 2014), Germany (Müller 1999), Hungary (Papp 2001), Poland (Draber-Monko 1991) and The Netherlands (Beuk 2001).

**Table 2.** Systematic overview of host-parasite associations recorded for Slovakian Hippoboscidae.

Parasite sp.	Hosts	Order	Family	Species
* Hippobosca equina *	Aves	Accipitriformes	Accipitridae	* Accipiter gentilis *
	Mammalia	Carnivora	Canidae	* Canis familiaris *
		Cetartiodactyla	Bovidae	* Bos taurus *
		Cetartiodactyla	Cervidae	* Capreolus capreolu *
		Perissodactyla	Equidae	* Equus caballus *
		Primates	Hominidae	* Homo sapiens *
* Hippobosca longipennis *	Mammalia	Carnivora	Canidae	* Canis familiaris *
* Hippobosca variegata *	Mammalia	Carnivora	Canidae	* Canis familiaris *
* Lipoptena cervi *	Aves	Passeriformes	Turdidae	* Turdus philomelos *
	Mammalia	Cetartiodactyla	Bovidae	*Caprahircus, Rupicaprarupicapra*
		Cetartiodactyla	Cervidae	*Capreoluscapreolus, Cervuselaphus*
		Primates	Hominidae	* Homo sapiens *
* Lipoptena fortisetosa *	Mammalia	Cetartiodactyla	Cervidae	* Capreolus capreolus *
		Primates	Hominidae	* Homo sapiens *
* Melophagus ovinus *	Mammalia	Carnivora	Canidae	* Canis familiaris *
		Cetartiodactyla	Bovidae	* Ovis aries *
		Perissodactyla	Equidae	* Equus caballus *
		Primates	Hominidae	* Homo sapiens *
* Melophagus rupicaprinus *	Mammalia	Cetartiodactyla	Bovidae	*Ovisaries, Rupicaprarupicapra*
* Crataerina pallida *	Aves	Apodiformes	Apodidae	* Apus apus *
* Icosta ardeae *	Aves	Passeriformes	Hirundinidae	* Ardea purpurea *
	Aves	Pelecaniformes	Ardeidae	* Ixobrychus minutus *
* Icosta minor *	Aves	Passeriformes	Passeridae	* Passer montanus *
* Ornithoica turdi *	Aves	Passeriformes	Emberizidae	* Emberiza citrinella *
	Aves		Fringillidae	* Fringilla coelebs *
	Aves		Sittidae	* Sitta europaea *
* Ornithophila metallica *	Aves	Passeriformes	Muscicapidae	* Saxicola rubetra *
* Pseudolynchia canariensis *	Aves	Accipitriformes	Pandionidae	* Pandion halieaetus *
* Stenepteryx hirundinis *	Aves	Passeriformes	Hirundinidae	*Delichon urbica, Hirundorustica*
* Ornithomya avicularia *	Aves	Accipitriformes	Accipitridae	*Accipitergentilis, Aquilapomarina, Circuspygargus, Pernisapivorus*
		Anseriformes	Anatidae	* Anser anser *
		Falconiformes	Falconidae	* Falco tinnunculus *
		Passeriformes	Prunellidae	* Prunella modularis *
			Acrocephalidae	*Acrocephalusarundinaceus, A.melanopogon, A.scirpaceus*
			Corvidae	* Pica pica *
			Emberizidae	* Emberiza cia *
			Fringillidae	*Carduelischloris, Coccothraustescoccothraustes, Fringillacoelebs*
			Laniidae	*Laniuscollurio, L.excubitor*
			Locustellidae	* Locustella luscinioides *
			Motacillidae	* Anthus trivialis *
			Muscicapidae	*Erithacusrubecula, Ficedulaalbicollis, Phoenicurusochruros, Saxicolarubetra*
			Oriolidae	* Oriolus oriolus *
			Paradoxornithidae	* Panurus biarmicus *
			Passeridae	*Passerdomesticus, P. montanus*
			Turdidae	*Monticolasaxatilis, Turdusmerula, T. pilaris*
		Strigiformes	Strigidae	* Strix aluco *
	Mammalia	Primates	Hominidae	* Homo sapiens *
* Ornithomya biloba *	Aves	Passeriformes	Hirundinidae	*Hirundorustica, Ripariariparia*
* Ornithomya chloropus *	Aves	Passeriformes	Sylviidae	* Regulus regulus *
* Ornithomya fringillina *	Aves	Passeriformes	Acrocephalidae	*Acrocephalusarundinaceus, A.schoenobaenus*
			Paridae	* Parus caeruleus *
			Hirundinidae	* Riparia riparia *
			Sittidae	* Sitta europaea *
			Sylviidae	* Sylvia atricapilla *
			Troglodytidae	* Troglodytes troglodytes *
		Pelecaniformes	Ardeidae	* Ardea purpurea *

The comparison of species richness of Hippoboscidae across Europe’s best studied countries for Diptera surprisingly showed that in Slovakia, the fauna of this group is not only distinctly more diverse than in all surrounding Central European countries, but even comparable with the faunas of much larger and more southern countries, such as Spain or Italy (including their insular areas), which also comprise 19 species (Table 1). However, raising any hypotheses about a possible latitudinal pattern in hippoboscid species richness would require much more thorough data on the continental scale.

We have noted that a number of country occurrences are missing in Fauna Europaea (Petersen 2004, Pape et al. 2015) when compared with the above checklists, most markedly for Spain and its adjacent islands (seven species missing) and Great Britain (six species missing). In contrast, some species listed in Fauna Europaea are missing in national checklists: e.g., *I.minor* is present in Fauna Europaea for Italy (Petersen 2004, Pape et al. 2015) but is absent in the national checklist (Pape et al. 1995); similarly, *O.chloropus* is present in Fauna Europaea for Hungary but is missing in the checklist of this country (Papp 2001). The national checklist of Spain (Carles-Tolrá and Báez 2002) also includes the species *Crataerinanigriventris* Gil Collado, 1932, which was wrongly referred to as *C.nigriventris* (Strobl, 1906) although it was originally described by Gil Collado (1932). However, according to Schneider-Orelli (1937), it is only an aberrant form of *C.melbae* (Rondani, 1879) and, therefore, it is omitted from the list in Table 1.

Judging from the occurrences of Hippoboscidae in other European countries, the list of Slovak species of the family is obviously not yet complete, despite its richness. At least the following two species can be expected in Slovakia: *Crataerinamelbae* and *Pseudolynchiagarzettae* (Rondani, 1879), both of which parasitize bird species living in Slovakia and are known from Italy and Switzerland, and Great Britain and Italy, respectively (see Table 1). In addition, the introduction of additional, more exotic hippoboscid species, like *Olfersiaspinifera* (Leach, 1817) (known from G. Britain) or *Ornithomyarupes* Hutson, 1981 (recorded from Switzerland) to Slovakia cannot be excluded. In conclusion, more than 20 species of louse flies can be expected to occur in Slovakia, including both native residents and sporadic introductions.

Several species of the family Hippoboscidae can interfere with human life and interests, as ectoparasites of some domestic animals, occasionally parasitizing also humans. These are mainly *Lipoptenacervi*, *L.fortisetosa*, and relatively rare species *Hippoboscaequina* and *Melophagusovinus*. Damage is caused by direct bloodsucking and the venomous saliva of the louse flies, which can lead to permanent loss of blood and to animal wasting (especially ovine wasting), reduced milk and wool production, damage to wool caused by the parasite’s faeces, etc. (Hutyra and Marek 1952). A secondary consequence of ectoparasitism by keds is the constant discomfort and scratching by the parasitised host (Hase 1927). Louse flies are also known as possible vectors of various diseases (Baker 1967, Kečera 1983, Oyieke and Reid 2003, Halos et al. 2004, Reeves et al. 2006, Martinković et al. 2012). A few of the above-mentioned species, especially *L.cervi* and *L.fortisetosa*, may bite humans in forest environments.

In Slovakia, species of the family Hippoboscidae have not received sufficient attention, even though they are among the most abundant ectoparasites in some localities. Therefore, it is important to pay attention to this group and maintain an accurate overview of the species living in our territory, including monitoring of the occurrence of non-native species migrating with their hosts or imported with domestic animals. From a wider perspective, verified and accurate information on the diversity and distribution of louse flies in Slovakia can contribute to knowledge of this parasitic group from a global point of view.
